# Supplementary figures and images for: The First Steps of Adaptation of Escherichia coli to the Gut Are Dominated by Soft Sweeps
Source: PLoS Genet. 2014 Mar 6;10(3):e1004182. doi: 10.1371/journal.pgen.1004182 (PMC3945185; doi:10.1371/journal.pgen.1004182)

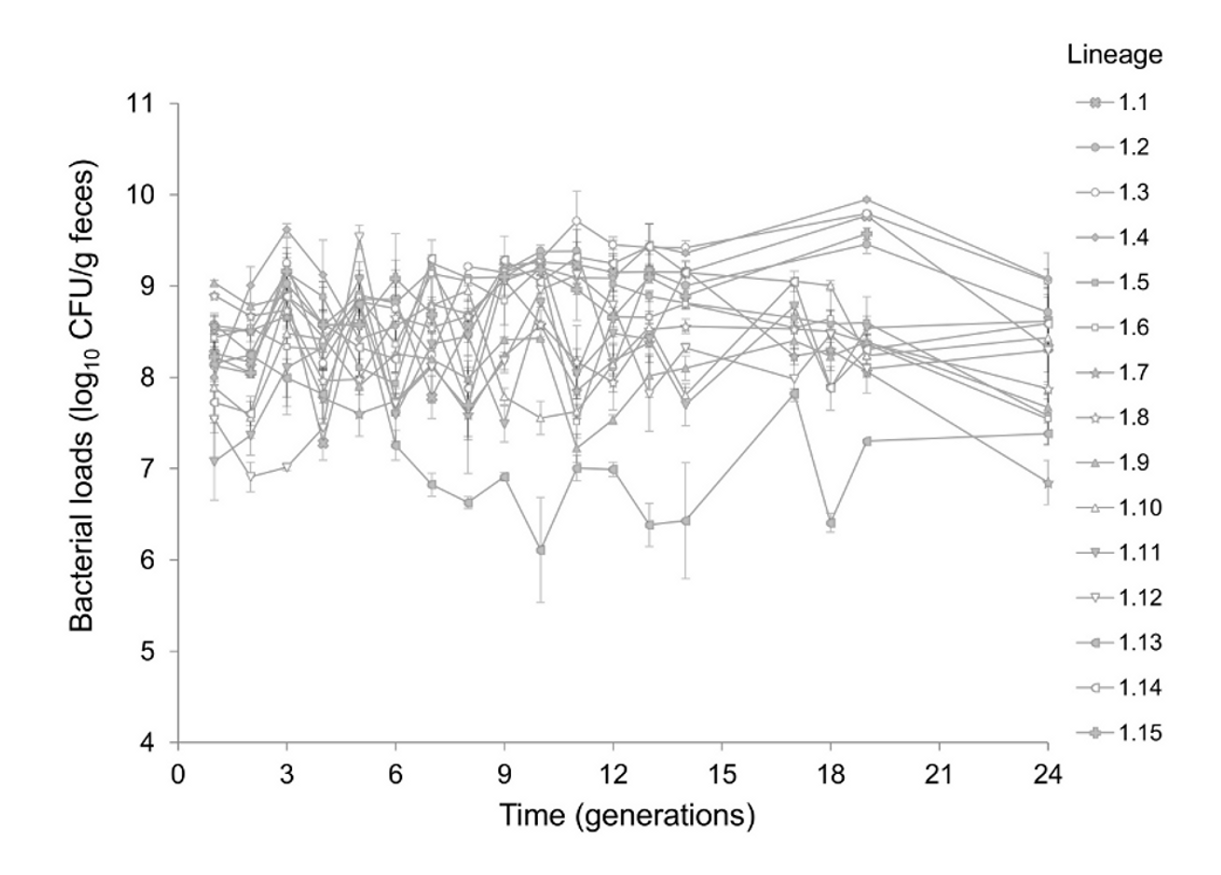

Supplement: Figure S1 — Colonization of the mouse gut by Escherichia coli. Bacterial loads per gram of feces (with 95% confidence intervals) during 24 days of adaptation of E. coli to the mouse gut (populations 1.1 to 1.15). (TIF) [file pgen.1004182.s001.tif]

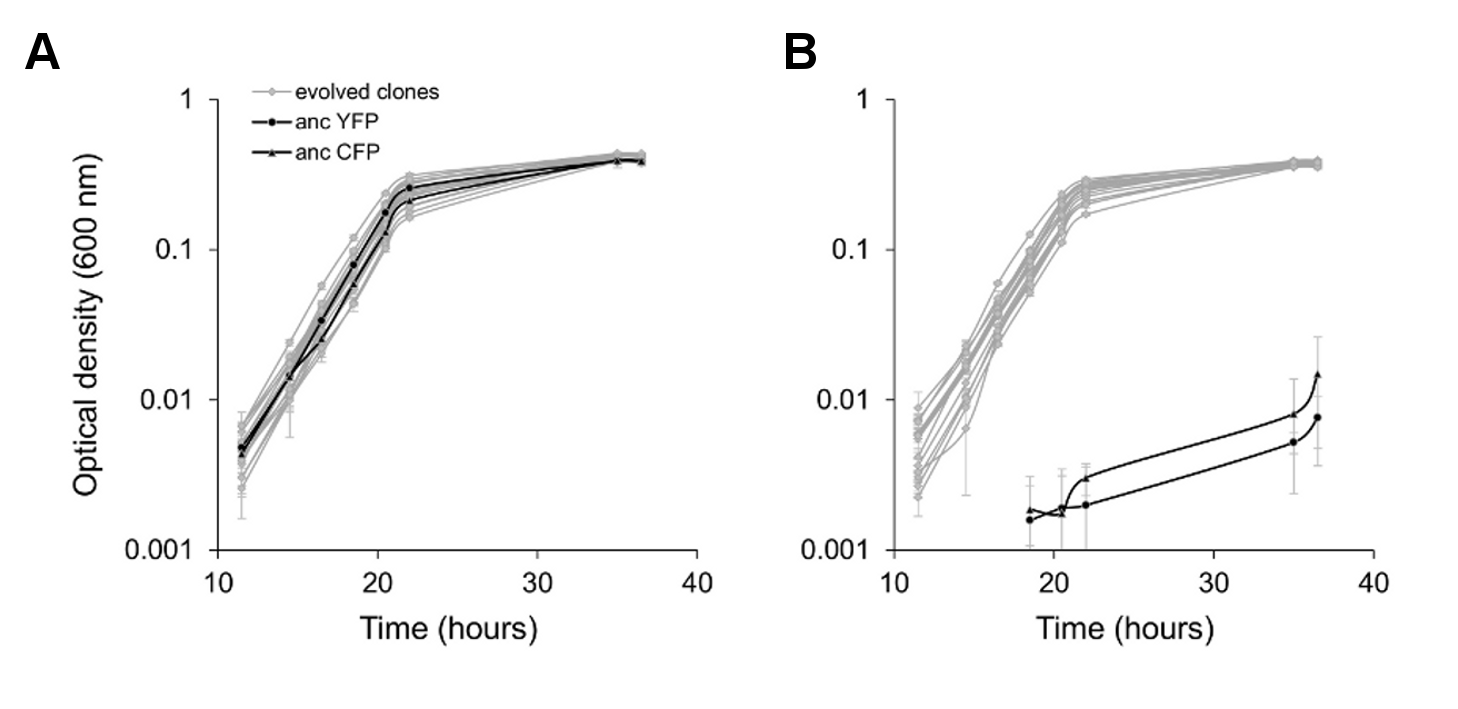

Supplement: Figure S2 — Comparison of growth curves of ancestral strain and evolved clones. Growth curves of ancestral (black) and evolved clones (grey) in MM with glycerol (A) and MM with glycerol and galactitol (B). Error bars represent the standard error of the mean of three independent measurements. (TIF) [file pgen.1004182.s002.tif]

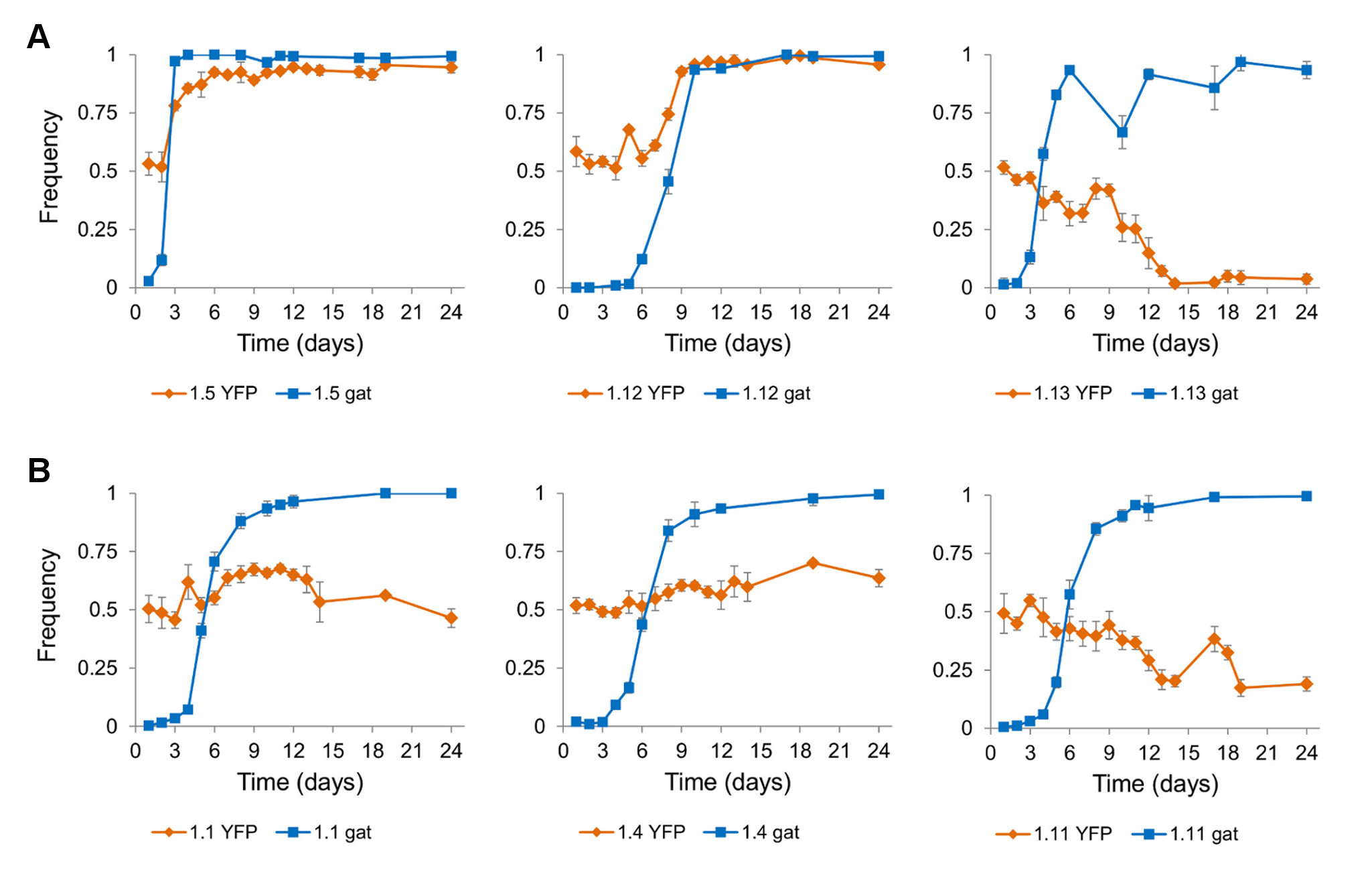

Supplement: Figure S3 — Emergence and spread of beneficial mutations in the gat operon. Dynamics of frequency change of the gat-negative phenotype (blue squares) and of the neutral fluorescent marker (orange diamonds) are shown for representative examples of populations where increase in frequency and eventual fixation of the gat-negative phenotype was accompanied by strong divergence of the fluorescent marker ((A) populations 1.5, 1.12 and 1.13) or not ((B) 1.1, 1.4 and 1.11). (TIF) [file pgen.1004182.s003.tif]

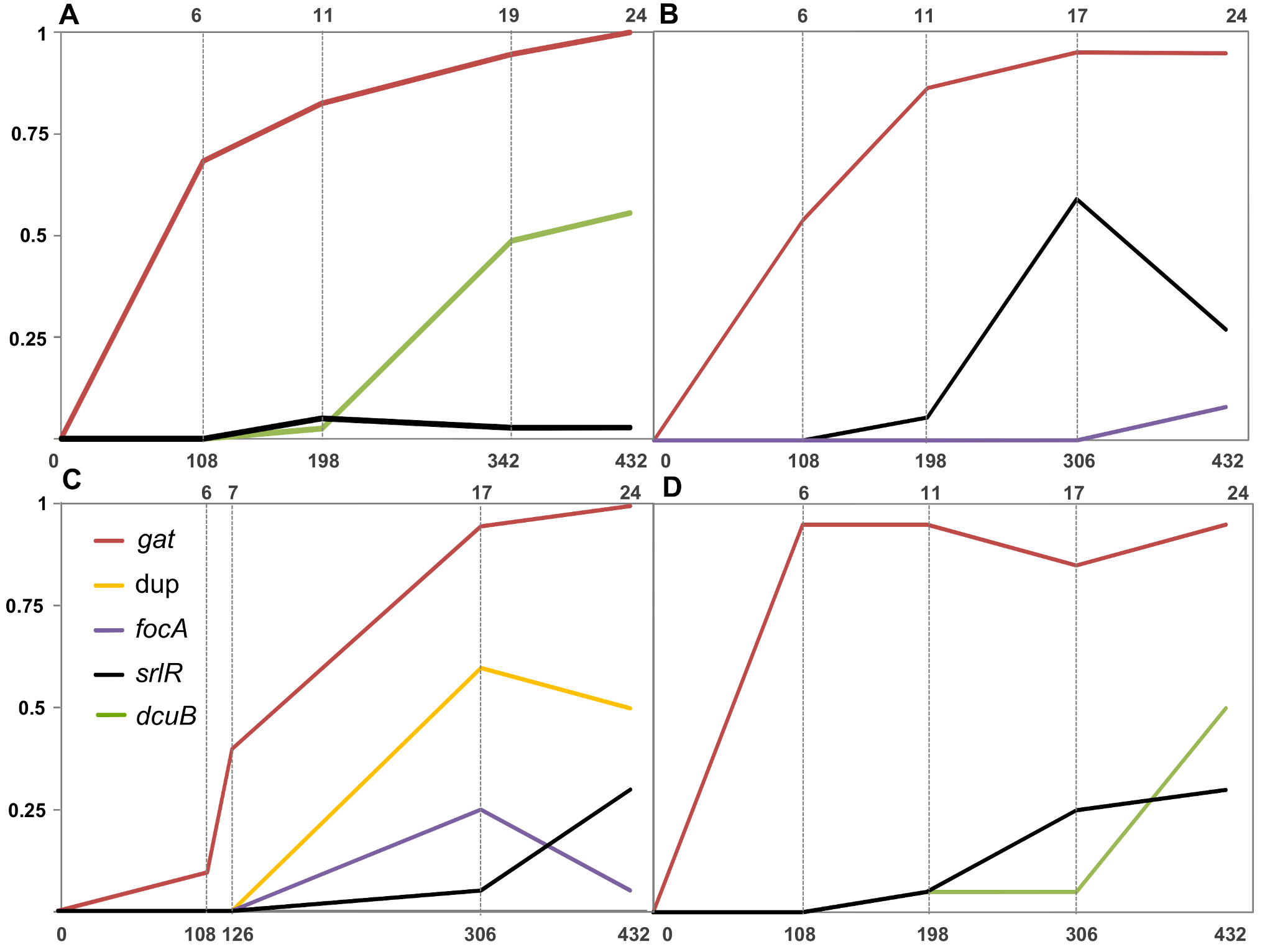

Supplement: Figure S5 — Sums of frequencies of genotypes at different loci along time for the populations represented in Figure 5. As in Figure 5 these frequencies are represented along 24 days (corresponding to 432 generations) of evolution inside the mouse gut (see Tables S3 to S6 for numeric data). A corresponds to population 1.1, B to 1.11, C to 1.12 and D to 1.5. (TIF) [file pgen.1004182.s005.tif]

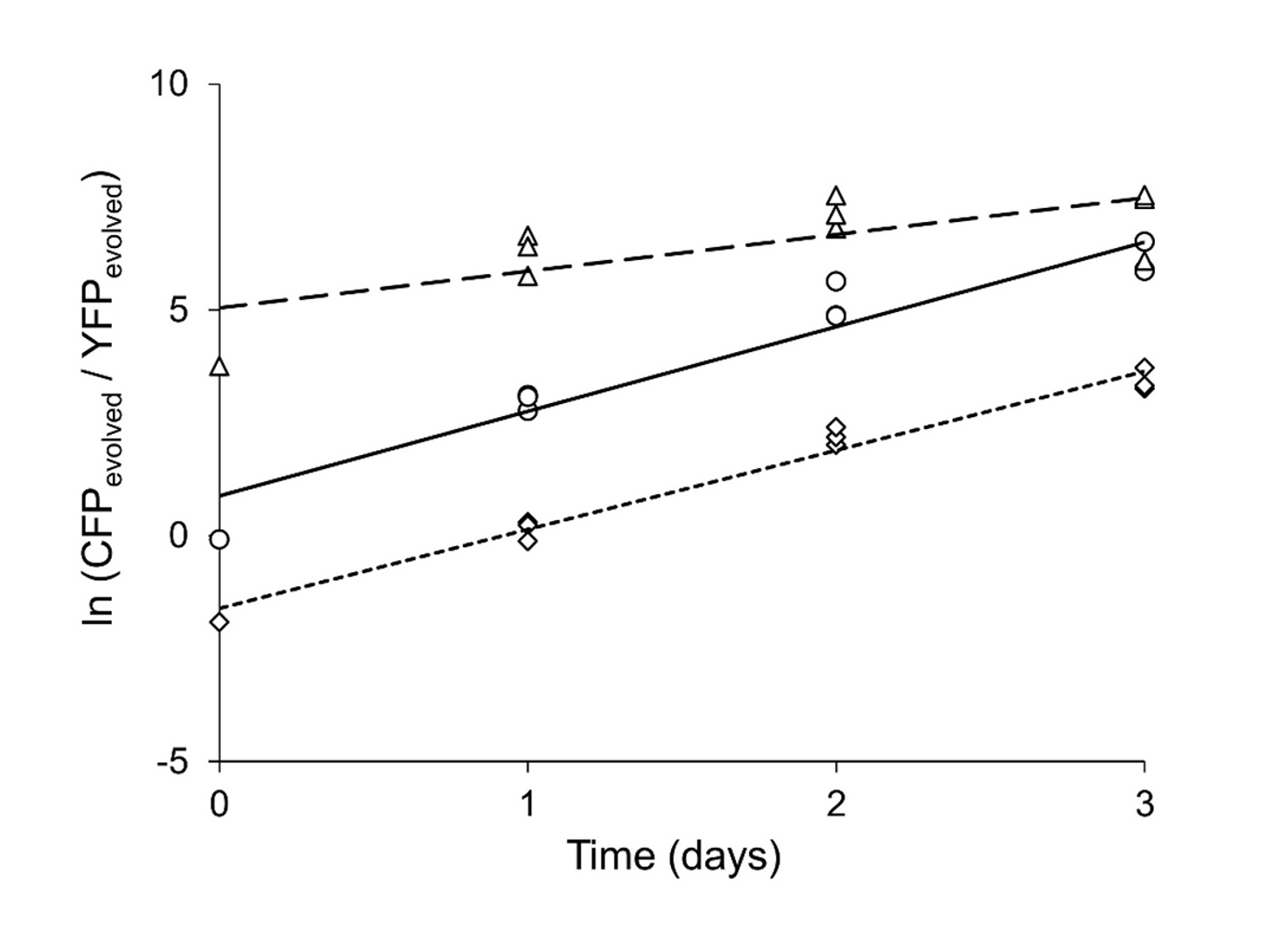

Supplement: Figure S7 — Test for negative frequency-dependent selection of evolved clones. 30 CFP and 30 YFP clones isolated from population 1.13 after 24 days of colonization were competed in vivo at initial ratios CFP:YFP of 1∶1 (open circles), 1∶10 (open diamonds) and 100∶1 (open triangles) for 3 days (corresponding to approximately 54 generations). Three independent competition experiments were performed for each ratio. The selective advantages per generation of the CFP in relation to the YFP population were calculated for each ratio of CFP over YFP. These are based on the slopes of the linear regression of ln(CFP/YFP) along time. The slopes (±2 s.e.m) are: 0.11 (±0.04), R2 = 0.95 for 1∶1 (dotted line); 0.10 (±0.02), R2 = 0.99 for 1∶10 (solid line) and 0.06 (±0.05), R2 = 0.77 for 100∶1(dashed line). A selective advantage was found for all the cases tested irrespective of their initial frequency, indicating that negative frequency-dependent selection is not the major process underling adaptation in the mouse gut. (TIF) [file pgen.1004182.s007.tif]
