# Supplementary material for: The First Steps of Adaptation of Escherichia coli to the Gut Are Dominated by Soft Sweeps
Source: PLoS Genet. 2014 Mar 6;10(3):e1004182. doi: 10.1371/journal.pgen.1004182 (PMC3945185; doi:10.1371/journal.pgen.1004182)
Supplement: Table S1 — Mutations identified in the genomes of the ancestral strain. (DOC) [file pgen.1004182.s009.doc]

**Table S1. Mutations identified in the genomes of the ancestral strain.**

Mutations were identified in the genome of the ancestor 0YFP by comparison with the reference genome [63]. Mutations in intergenic regions have the two flanking genes listed (e.g., fdrA/ylbF). Genes within brackets mean that the mutation happened within the gene. SNPs are represented by an arrow between the ancestral and the evolved nucleotide. Whenever a SNP gives rise to a non-synonymous mutation the amino acid replacement is also indicated. The symbol Δ means a deletion event and a + symbol represents an insertion of the nucleotide that follows the symbol. For intergenic mutations, the numbers in the annotation row represent nucleotides relative to each of the neighboring genes, where + indicates the distance downstream of the stop codon of a gene and - indicates the distance upstream of the gene, that is relative to the start codon. The mutations present in the 0YFP but not in the 0CFP, are underlined.

| **Clone** | **Genome Position** | **Gene** | **Mutation** | **Annotation** |
| --- | --- | --- | --- | --- |
| **0YFP** | 547694 | *fdrA/ylbF* | A→G | intergenic (+123/‑1156) |
|  | 547835 | *fdrA/ylbF* | +G | intergenic (+264/‑1015) |
|  | 1395405 | *[ynaJ]–[ttcA]* | Δ13,756 bp | multigenic |
|  | 1976527 | *insB–insA* | Δ776 bp |  |
|  | 2369558 | *arnT* | 4 bp x 2 | duplication |
|  | 3422257 | *rrlD* | A→C | noncoding |
|  | 3422258 | *rrlD* | T→A | noncoding |
|  | 3422259 | *rrlD* | C→T | noncoding |
|  | 3434719 | *trkA* | G→A | E60E GAG→GAA |
|  | 3472447 | *rpsL* | T→C | K43R AAA→AGA |
|  | 3844290 | *uhpT* | A→C | F301V TTT→GTT |
|  | 3957957 | *ppiC/rep* | C→T | intergenic (‑121/‑743) |
|  | 4095684 | *rhaB/rhaS* | T→C | intergenic (‑213/‑75) |
|  | 360473 | *lacA–lacI* | Δ6264 bp | multigenic |
|  | 788169 | *[galK]* | Δ1034 bp |  |
|  | 4294082 | *RIP321* | Δ338 bp |  |
