# Supplementary material for: The First Steps of Adaptation of Escherichia coli to the Gut Are Dominated by Soft Sweeps
Source: PLoS Genet. 2014 Mar 6;10(3):e1004182. doi: 10.1371/journal.pgen.1004182 (PMC3945185; doi:10.1371/journal.pgen.1004182)
Supplement: Table S2 — The number and nature of adaptive events across independently evolved clones. (DOC) [file pgen.1004182.s010.doc]

**Table S2. The number and nature of adaptive events across independently evolved clones.**

In the list of mutations, the initials IS denote the abbreviation of insertion sequence element at the indicated position. The asterisk means that the corresponding SNP originated a STOP codon. For further details see Table S1 legend. The last column shows the number of mutations segregating in the lineage from which the sequenced clone was isolated, as inferred from the theoretical model.

| **Clone** | **Genome Position** | **Gene** | **Mutation** | **Annotation** | **Inferred mutations** |
| --- | --- | --- | --- | --- | --- |
| **1CFP** | 2173589 | *gatZ* | IS Ins | coding (755/1263) | 2 |
|  | 4346888 | *dcuB/dcuR* | IS Ins | intergenic (-121/+450) |
| **2YFP** | 2173759 | *gatZ* | IS Ins | coding (585/1263) | 2 |
|  | 4346888 | *dcuB/dcuR* | IS Ins | intergenic (‑121/+450) |
| **3YFP** | 2560011 | *yffN* | G→A | C122Y TGC→TAC | 2 |
|  | 2175242 | *gatY/fbaB* | IS Ins | intergenic (‑16/+292) |
|  | 4601260 | *yjjP/yjjQ* | IS Ins | intergenic (‑379/‑244) |
| **4YFP** | 2173531 | *gatZ* | IS1 Ins | coding (813/1263) | 3 |
| **5YFP** | 2827493 | *srlR* | G→C | G142A GGC→GCC | 2 |
|  | 2172869 | *gatA* | IS Ins | coding (203/453) |
| **6YFP** | 2172262 | *gatC* | del 1 bp | coding (39/1356) | 2 |
|  | 4346888 | *dcuB/dcuR* | IS Ins | intergenic (‑121/+450) |
| **7YFP** | 1420379 | *ydaV* | C→A | L125M CTG→ATG | 4 |
|  | 1902231 | *[manZ]–[kdgR]* | Δ5,451 bp |  |
|  | 2175298 | *gatY/fbaB* | IS Ins | intergenic (-72/+236) |
|  | 953904 | *focA/ycaO* | IS Ins | intergenic (-212/+194) |
| **8YFP** | 3268729 | *garK* | A→G | F355S TTC→TCC | 2 |
|  | 2172869 | *gatA* | IS Ins | coding (203/453) |
| **9YFP** | 2827073 | *srlR* | A→T | K2I AAA→ATA | 2 |
|  | 2172636 | *gatA* | IS Ins | coding (436/453) |
| **10CFP** | 2174223 | *gatZ* | Δ2 bp | coding (120‑121/1263) | 4 |
|  | 1388754 | *[ycjY-ynaI]* | Δ5315 bp | large deletion |
|  | 953904 | *focA/ycaO* | IS5 Ins | intergenic (-212/+194) |
| **11CFP** | 2827095 | *srlR* | Δ1 bp | coding (27/774) | 4 |
|  | 2172869 | *gatA* | IS Ins | coding (203/453) |
| **12YFP** | 2172079 | *gatC* | +C | coding (222/1356) | 3 |
|  | 4500113 | *[insG-yaaI]* | 2x 151716 bp | large duplication |
| **13CFP** | 4637714 | *arcA* | G→T | R206S CGC→AGC | 2 |
|  | 2171153 | *gatC* | IS Ins | coding (1148/1356) |
| **14CFP** | 943941 | *dmsC* | G→A | W229* TGG→TAG | 2 |
|  | 2175263 | *gatY/fbaB* | IS Ins | intergenic (-37/+271) |
|  | 2827117 | *srlR* | C→T | Q17* CAG→TAG |
