# Supplementary material for: The First Steps of Adaptation of Escherichia coli to the Gut Are Dominated by Soft Sweeps
Source: PLoS Genet. 2014 Mar 6;10(3):e1004182. doi: 10.1371/journal.pgen.1004182 (PMC3945185; doi:10.1371/journal.pgen.1004182)
Supplement: Table S3 — Frequencies of newly generated haplotypes along 24 days of evolution of population 1.1 inside the mouse gut. (DOCX) [file pgen.1004182.s011.docx]

**Table S3. Frequencies of newly generated haplotypes along 24 days of evolution of population 1.1 inside the mouse gut.**

Yellow and blue shading identify haplotypes belonging to the two sub-populations of bacteria labeled either with *cfp* or *yfp* alleles. The ancestral haplotype is, by definition, devoid of mutations. The genome position for a given mutation is only indicated for mutations that are not IS insertions. Mutations in intergenic regions have the two flanking genes listed (e.g., *dcuB/dcuR*). Whenever a SNP gives rise to a non-synonymous mutation the amino acid replacement is indicated. Synonymous mutations are represented as Syn. The symbol Δ means a deletion event and a + symbol represents an insertion of the nucleotide that follows the symbol. The asterisk means that the corresponding SNP originated a STOP codon. IS insertions at given position are indicated as IS Ins. The exact location of IS insertions is not indicated since it was determined (see Material and Methods), however the detection strategy allowed us to distinguish between different insertions in the same gene. Therefore every haplotype indicated in Table S3 was confirmed to be unique. See Figure 5 for a graphical representation of the data in this table.

| **Genome Position** | **Gene** | **Mutation** | **Haplotype frequencies** | | | | |
| --- | --- | --- | --- | --- | --- | --- | --- |
|  |  |  | **0 gen** | **108 gen** | **198 gen** | **342 gen** | **432 gen** |
|  |  |  | 0.5 | 0.17 | 0.10 |  |  |
|  | *dcuB/dcuR* | IS Ins |  |  |  | 0.03 |  |
|  | *gatZ* | IS Ins |  | 0.07 | 0.05 |  | 0.03 |
|  | *gatZ* | IS Ins |  |  | 0.03 | 0.30 | 0.42 |
|  | *dcuB/dcuR* | IS Ins |  |  |  |  |  |
|  | *gatZ* | IS Ins |  |  |  | 0.03 |  |
|  | *dcuB/dcuR* | IS Ins |  |  |  |  |  |
| 2827852 | *srlR* | Δ 1bp |  |  |  |  |  |
| 2173887 | *gatZ* | Δ 5bp |  | 0.02 |  |  |  |
| 2171894 | *gatC* | Δ 1bp |  |  | 0.03 |  |  |
|  | *gatY* | IS Ins |  | 0.02 | 0.03 |  | 0.06 |
|  | *gatY* | IS Ins |  |  |  | 0.03 |  |
|  | *dcuB/dcuR* | IS Ins |  |  |  |  |  |
|  | *gatC* | IS Ins |  | 0.02 | 0.03 |  |  |
|  | *gatA* | IS Ins |  | 0.05 | 0.08 |  |  |
|  | *gatA* | IS Ins |  | 0.05 | 0.03 |  |  |
|  |  |  | 0.5 | 0.15 | 0.08 | 0.03 |  |
|  | *gatZ* | IS Ins |  | 0.12 | 0.38 | 0.46 | 0.28 |
|  | *gatZ* | IS Ins |  |  | 0.03 |  |  |
| 2827549 | *srlR* | FE161QK |  |  |  |  |  |
|  | *gatZ* | IS Ins |  |  |  | 0.05 | 0.03 |
|  | *dcuB/dcuR* | IS Ins |  |  |  |  |  |
|  | *gatY* | IS Ins |  | 0.02 |  |  | 0.06 |
|  | *gatC* | IS Ins |  | 0.05 | 0.05 |  |  |
| 2171454 | *gatC* | Q155* |  |  | 0.03 |  |  |
| 2172118 | *gatC* | +C |  |  |  | 0.03 |  |
| 2174029 | *gatZ* | ∆1bp |  | 0.02 |  |  |  |
| 2173899 | *gatZ* | Δ 5bp |  | 0.02 | 0.05 |  |  |
| 2173900 | *gatZ* | Δ 5bp |  | 0.12 |  |  |  |
| 2173900 | *gatZ* | Δ 5bp |  |  |  | 0.05 | 0.08 |
|  | *dcuB/dcuR* | IS Ins |  |  |  |  |  |
|  | *gatA* | IS Ins |  | 0.05 |  |  |  |
|  | *gatA* | IS Ins |  | 0.02 | 0.03 |  |  |
|  | *gatA* | IS Ins |  |  |  |  | 0.03 |
|  | *dcuB/dcuR* | IS Ins |  |  |  |  |  |
|  | *gatA* | IS Ins |  |  | 0.03 |  | 0.03 |
| 2827529 | *srlR* | G154E |  |  |  |  |  |
