# Supplementary material for: The First Steps of Adaptation of Escherichia coli to the Gut Are Dominated by Soft Sweeps
Source: PLoS Genet. 2014 Mar 6;10(3):e1004182. doi: 10.1371/journal.pgen.1004182 (PMC3945185; doi:10.1371/journal.pgen.1004182)
Supplement: Table S4 — Frequencies of newly generated haplotypes along 24 days of evolution of population 1.5 inside the mouse gut. (DOCX) [file pgen.1004182.s012.docx]

**Table S4.** **Frequencies of newly generated haplotypes along 24 days of evolution of population 1.5 inside the mouse gut.**

See Table S3 for further details.

| **Genome Position** | **Gene** | **Mutation** | **Haplotype frequencies** | | | | |
| --- | --- | --- | --- | --- | --- | --- | --- |
|  |  |  | **0 gen** | **108 gen** | **198 gen** | **306 gen** | **432 gen** |
|  |  |  | 0.5 |  |  | 0.05 |  |
|  | *dcuB/dcuR* | IS Ins |  |  |  |  | 0.05 |
|  | *gatA* | IS Ins |  | 0.05 |  |  |  |
|  | *gatA* | IS Ins |  | 0.10 |  | 0.10 |  |
|  | *gatA* | IS Ins |  |  | 0.05 |  |  |
|  | *dcuB/dcuR* | IS Ins |  |  |  |  |  |
|  | *gatY/fbaB* | IS Ins |  | 0.05 | 0.05 |  | 0.05 |
|  |  |  | 0.5 | 0.05 | 0.05 | 0.10 |  |
|  | *gatY* | IS Ins |  | 0.75 | 0.65 | 0.40 | 0.15 |
|  | *gatY* | IS Ins |  |  | 0.05 | 0.05 |  |
| 2827489 | *srlR* | P141S |  |  |  |  |  |
|  | *gatY* | IS Ins |  |  |  | 0.05 |  |
| 2827490 | *srlR* | P141L |  |  |  |  |  |
|  | *gatY* | IS Ins |  |  |  |  | 0.05 |
| 2827493 | *srlR* | G142E |  |  |  |  |  |
|  | *gatY* | IS Ins |  |  |  |  | 0.45 |
|  | *dcuB/dcuR* | IS Ins |  |  |  |  |  |
|  | *gatA* | IS Ins |  |  | 0.15 | 0.05 |  |
|  | *gatA* | IS Ins |  |  |  | 0.15 | 0.25 |
| 2827493 | *srlR* | G142A |  |  |  |  |  |
|  | *gatA* | IS Ins |  |  |  | 0.05 |  |
|  | *dcuB/dcuR* | IS Ins |  |  |  |  |  |
