# Supplementary material for: The First Steps of Adaptation of Escherichia coli to the Gut Are Dominated by Soft Sweeps
Source: PLoS Genet. 2014 Mar 6;10(3):e1004182. doi: 10.1371/journal.pgen.1004182 (PMC3945185; doi:10.1371/journal.pgen.1004182)
Supplement: Table S5 — Frequencies of newly generated haplotypes along 24 days of evolution of population 1.11 inside the mouse gut. (DOCX) [file pgen.1004182.s013.docx]

**Table S5** **– Frequencies of newly generated haplotypes along 24 days of evolution of population 1.11 inside the mouse gut.**

See Table S3 for further details.

| **Genome Position** | **Gene** | **Mutation** | **Haplotype frequencies** | | | | |
| --- | --- | --- | --- | --- | --- | --- | --- |
|  |  |  | **0 gen** | **108 gen** | **198 gen** | **306 gen** | **432 gen** |
|  |  |  | 0.5 | 0.23 | 0.08 |  |  |
|  | *gatA* | IS Ins |  | 0.33 | 0.50 | 0.15 | 0.57 |
|  | *gatA* | IS Ins |  |  | 0.03 | 0.08 | 0.03 |
| 2827492 | *srlR* | G142S |  |  |  |  |  |
|  | *gatA* | IS Ins |  |  |  | 0.03 |  |
| 2827493 | *srlR* | G142D |  |  |  |  |  |
|  | *gatA* | IS Ins |  |  |  | 0.03 |  |
| 2827728 | *srlR* | del1bp |  |  |  |  |  |
|  | *gatA* | IS Ins |  |  |  | 0.18 | 0.03 |
| 2827177 | *srlR* | del7bp |  |  |  |  |  |
|  | *gatA* | IS Ins |  |  |  | 0.03 |  |
| 2827627 | *srlR* | E187K |  |  |  |  |  |
|  | *gatA* | IS Ins |  |  |  | 0.03 |  |
| 2827076 | *srlR* | P3L |  |  |  |  |  |
|  | *gatA* | IS Ins |  |  |  | 0.03 |  |
| 2827726 | *srlR* | ins7bp |  |  |  |  |  |
|  | *gatA* | IS Ins |  |  |  |  | 0.03 |
| 2827764 | *srlR* | Syn |  |  |  |  |  |
|  | *gatA* | IS Ins |  |  |  |  | 0.05 |
|  | *focA* | IS Ins |  |  |  |  |  |
|  | *gatA* | IS Ins |  |  |  |  | 0.03 |
| 2827492 | *srlR* | G142S |  |  |  |  |  |
|  | *focA* | IS Ins |  |  |  |  |  |
| 2173900 | *gatZ* | Δ 5bp |  | 0.05 | 0.03 | 0.03 |  |
|  | *gatC* | IS Ins |  | 0.03 |  |  |  |
|  | *gatY* | IS Ins |  |  | 0.03 |  |  |
|  | *gatZ* | IS Ins |  |  | 0.03 |  |  |
|  |  |  | 0.5 | 0.23 | 0.06 | 0.05 | 0.05 |
| 2173878 | *gatZ* | Δ 5bp |  | 0.03 | 0.03 |  |  |
| 2173878 | *gatZ* | Δ 5bp |  |  |  | 0.03 |  |
| 2827278 | *srlR* | del2bp |  |  |  |  |  |
| 2173878 | *gatZ* | Δ 5bp |  |  |  | 0.03 |  |
| 2827492 | *srlR* | G142S |  |  |  |  |  |
|  | *gatC* | IS Ins |  | 0.08 | 0.11 |  |  |
|  | *gatA* | IS Ins |  |  | 0.06 | 0.03 |  |
|  | *gatA* | IS Ins |  |  |  | 0.03 |  |
| 2827394 | *srlR* | L109P |  |  |  |  |  |
|  | *gatA* | IS Ins |  |  |  |  | 0.05 |
| 2827496 | *srlR* | G143V |  |  |  |  |  |
|  | *gatA* | IS Ins |  | 0.03 |  | 0.03 | 0.03 |
|  | *gatA* | IS Ins |  |  |  | 0.03 |  |
| 2827664 | *srlR* | A99E |  |  |  |  |  |
|  | *gatA* | IS Ins |  |  |  |  | 0.03 |
| 2827529 | *srlR* | G154E |  |  |  |  |  |
| 2174150 | *gatY* | del total |  |  | 0.03 | 0.13 | 0.03 |
| 2174150 | *gatY* | del total |  |  | 0.03 | 0.10 | 0.03 |
| 2827172 | *srlR* | T35N |  |  |  |  |  |
|  | *gatY* | IS Ins |  |  |  |  | 0.03 |
| 2827172 | *srlR* | T35N |  |  |  |  |  |
|  | *gatZ* | IS Ins |  |  |  |  | 0.03 |
| 2827276 | *srlR* | Δ 2bp |  |  |  |  |  |
