# Supplementary material for: The First Steps of Adaptation of Escherichia coli to the Gut Are Dominated by Soft Sweeps
Source: PLoS Genet. 2014 Mar 6;10(3):e1004182. doi: 10.1371/journal.pgen.1004182 (PMC3945185; doi:10.1371/journal.pgen.1004182)
Supplement: Table S6 — Frequencies of newly generated haplotypes along 432 generations of evolution of population 1.12 inside the mouse gut. (DOCX) [file pgen.1004182.s014.docx]

**Table S6. Frequencies of newly generated haplotypes along 24 days of evolution of population 1.12 inside the mouse gut.**

See Table S3 for further details.

| **Genome Position** | **Gene** | **Mutation** | **Haplotype frequencies** | | | | |
| --- | --- | --- | --- | --- | --- | --- | --- |
|  |  |  | **0 gen** | **108 gen** | **126 gen** | **306 gen** | **432 gen** |
|  |  |  | 0.5 | 0.48 | 0.20 | 0.05 |  |
| 2172078 | *gatC* | +C |  |  |  | 0.05 |  |
|  |  |  | 0.5 | 0.42 | 0.40 |  |  |
|  | *gatC* | +C |  | 0.09 | 0.40 |  | 0.15 |
|  | *gatC* | +C |  |  |  | 0.60 | 0.50 |
|  | *dup* |  |  |  |  |  |  |
|  | *gatC* | +C |  |  |  | 0.25 | 0.05 |
|  | *focA* | IS Ins |  |  |  |  |  |
|  | *gatC* | +C |  |  |  | 0.05 | 0.05 |
| 2827117 | *srlR* | Q17* |  |  |  |  |  |
|  | *gatC* | +C |  |  |  |  | 0.25 |
| 2827234 | *srlR* | L97V |  |  |  |  |  |
