# Supplementary material for: The First Steps of Adaptation of Escherichia coli to the Gut Are Dominated by Soft Sweeps
Source: PLoS Genet. 2014 Mar 6;10(3):e1004182. doi: 10.1371/journal.pgen.1004182 (PMC3945185; doi:10.1371/journal.pgen.1004182)
Supplement: Table S7 — The number and nature of adaptive events across independently evolved populations. (DOCX) [file pgen.1004182.s015.docx]

**Table S7. The number and nature of adaptive events across independently evolved populations.**

In the list of mutations del/dup denotes that either a deletion or a duplication of the indicated size occurred but it is not possible to distinguish between the two. For further details see Table S1 legend. Underlined mutations were also found in the sequenced clones isolated from the corresponding populations (see Table S2).

| **Population** | **Genome Position** | **Gene** | **Mutation** | **Annotation** |
| --- | --- | --- | --- | --- |
| **1.10** | 735886 | *ybfQ* | C→T | pseudogene (219/240 nt) |
|  | 1464225 | *ydbA* | T→C | pseudogene (1704/2513 nt) |
|  | 2174223 | *gatZ* | Δ2 bp | coding (120-121/1263 nt) |
|  | 2827492 | *srlR* | G→A | G142S (GGC→AGC) |
|  | 1706868 | *rsxC* | C→G | A642A (GCC→GCG) |
|  | 953904 | *focA/ycaO* | IS Ins |  |
|  | 953895 | *focA/ycaO* | IS Ins |  |
|  | 2171093 | *gatC* | IS Ins |  |
|  | 2172273 | *gatC* | IS Ins |  |
| **1.11** | 2172869 | *gatA* | IS Ins | coding (203/453 nt) |
|  | 2827172 | *srlR* | C→A | T35N (ACC→AAC) |
|  | 4601110 | *yjjP/yjjQ* | IS Ins | intergenic (‑229/‑387) |
|  | 2174150 | *[gat Z-gatY]* | Δ1111bp |  |
|  | 4601260 | *yjjP/yjjQ* | IS Ins |  |
|  | 3925441 | *asnA* | del/dup 40bp |  |
|  | 761046 | *sucB* | del/dup 67bp |  |
|  | 3996061 | *uvrD* | del/dup 39bp |  |
|  | 2176626 | *fbaB/yegT* | IS Ins |  |
|  | 2545378 | *murP* | del/dup 15bp |  |
| **1.12** | 2827234 | *srlR* | G→A | M56M (GTG→ATG) |
|  | 953904 | *focA/ycaO* | IS Ins |  |
|  | 3925441 | *asnA* | del/dup 39bp |  |
|  | 281295 | *yagA/yagE* | del/dup 35bp |  |
|  | 1284572 | *narJ* | del/dup 47bp |  |
|  | 2172079 | *gatC* | +C | coding (222/1356 nt) |
|  | 2440543 | *mnmC* | del/dup 106bp |  |
|  | 4570195 | *yjiS/yjiT* | IS Ins |  |
|  | 4500113 | *[insG-yaaI]* | 2x 151716 bp | large duplication |
