# Supplementary material for: The First Steps of Adaptation of Escherichia coli to the Gut Are Dominated by Soft Sweeps
Source: PLoS Genet. 2014 Mar 6;10(3):e1004182. doi: 10.1371/journal.pgen.1004182 (PMC3945185; doi:10.1371/journal.pgen.1004182)
Supplement: Table S8 — Oligonucleotide primers used in this work. (DOCX) [file pgen.1004182.s016.docx]

**Table S8. Oligonucleotide primers used in this work.**

| **Gene** | **Forward (5' - 3')** | **Reverse (5' - 3')** | **Usage** |
| --- | --- | --- | --- |
| ***dcuB*** | GGCTGAAGGTGGAAGACGAA | ACATTTCGCGTGTTTCCTGC | Amplification of *dcuB* |
| ***focA*** | AGCGGATGTTTCGTTGCTTT | TGCTGCACATCAGTCGTTGT | Amplification of *focA* |
| ***gatABCD*** | TCCCACCGCATCAATATAGCC | CAGTCCGGGGAATTATCAGCA | Amplification of *gatA, gatB, gatC and gatD* |
| ***gatZY*** | CACCTTTGGCGAGCATCTCA | AAAACACGCGCACTTTGCTA | Amplification of *gatZ and gatY* |
| ***gatB*** | GATCCACTTTGGCAGTGGTG | CAGTCCGGGGAATTATCAGCA | Amplification of *gatB* |
| ***gatY*** | GCCACAATCGGCAATCACTT | AAAACACGCGCACTTTGCTA | Amplification of *gatY* |
| ***dupl 150kb*** | GTTCGTTGCGCATCAGTACG | GCTCTACCCGCAGGTCAAAA | Amplification of the new junction |
| ***srlR*** | GCATGCGGGTGATTTACAGC | TTCCGGTAAACGGCTTGCTT | Amplification/sequencing of *srlR* |
| ***gatC*** | ATTAGCCGCCAGTTGGGTG |  | sequencing of gatC |
|  | TGCCGATAATCAGCCCCATC |  | sequencing of gatC |
|  | CCAGCCAACATCGACCACAT |  | sequencing of gatC |
| ***gatZ*** | ATATCGCCTCGCGTAAAGCA |  | sequencing of gatZ |
|  | ACCGTTTCTGGTGCTAACGG |  | sequencing of gatZ |
